# Study of Friction and Wear Effects in Aluminum Parts Manufactured via Single Point Incremental Forming Process Using Petroleum and Vegetable Oil-Based Lubricants

**DOI:** 10.3390/ma14143973

**Published:** 2021-07-16

**Authors:** José M. Diabb Zavala, Oscar Martínez-Romero, Alex Elías-Zúñiga, Héctor Manuel Leija Gutiérrez, Alejandro Estrada-de la Vega, Jaime Taha-Tijerina

**Affiliations:** 1 Facultad de Ingeniería Mecánica y Eléctrica (FIME), Universidad Autónoma de Nuevo León, FIME, Av. Universidad S/N, Ciudad Universitaria, 66451 San Nicolás de los Garza, NL, Mexico; jose.diabbzv@uanl.edu.mx; 2 School of Engineering and Science, Tecnologico de Monterrey, Av. E. Garza Sada 2501 Sur, 64849 Monterrey, NL, Mexico; alejandro_estrada@tec.mx; 3 Laboratorio de Nanociencias y Nanotecnología, Universidad Autónoma de Nuevo León, CICFM-FCFM, Av. Universidad S/N, Ciudad Universitaria, 66451 San Nicolás de los Garza, NL, Mexico; h.leija@gmail.com; 4 Engineering Department, Universidad de Monterrey, Av. Ignacio Morones Prieto 4500 Pte., 66238 San Pedro Garza García, NL, Mexico; jose.taha@udem.edu; 5 Department of Manufacturing and Industrial Engineering, University of Texas Rio Grande Valley, Brownsville, TX 78520, USA

**Keywords:** mineral and vegetable oils, friction and wear effects, single point incremental forming process, SPIFed Stribeck curve, surface roughness

## Abstract

This paper focuses on studying how mineral oil, sunflower, soybean, and corn lubricants influence friction and wear effects during the manufacturing of aluminum parts via the single point incremental forming (SPIF) process. To identify how friction, surface roughness, and wear change during the SPIF of aluminum parts, Stribeck curves were plotted as a function of the SPIF process parameters such as vertical step size, wall angle, and tool tip semi-spherical diameter. Furthermore, lubricant effects on the surface of the formed parts are examined by energy dispersive spectroscopy (EDS) and scanning electron microscope (SEM) images, the Alicona optical 3D measurement system, and Fourier-transform infrared spectroscopy (FTIR). Results show that during the SPIF process of the metallic specimens, soybean and corn oils attained the highest friction, along forces, roughness, and wear values. Based on the surface roughness measurements, it can be observed that soybean oil produces the worst surface roughness finish in the direction perpendicular to the tool passes (*Ra* =1.45 μm) considering a vertical step size of 0.25 mm with a 5 mm tool tip diameter. These findings are confirmed through plotting SPIFed Stribeck curves for the soybean and corn oils that show small hydrodynamic span regime changes for an increasing sample step-size forming process. This article elucidates the effects caused by mineral and vegetable oils on the surface of aluminum parts produced as a function of Single Point Incremental Sheet Forming process parameters.

## 1. Introduction

The single point incremental forming process (SPIF) is widely used to manufacture small batches of metallic and polymeric parts at room temperature using a CNC milling center, a support fixed to the metallic sheet to be formed, a spherical tip tool, and lubricants to reduce wear and friction force effects. In this sense, Allwood et al. [1] investigated the influence that through-thickness shear (TTS) has on the forming part limit by correlating the friction effects and the tool rotational speed during SPIF. Petek and co-workers studied the tribological effects that occur during the SPIF process [2]. They concluded that tool temperature is directly connected to SPIF parameters and friction conditions. Therefore, tribological properties can be improved during SPIF if coatings based on hard carbon are used on the tool tip. Hussain et al. addressed some relevant findings concerning the formability of commercially pure titanium. They concluded that if a proper tool and lubricants are used during the negative incremental forming of titanium parts, it is possible to form a part with good surface quality [3]. Later, with a small mathematical model, Durante et al. [4] investigated how SPIF parameters such as tool tip radius, vertical step depth, and wall angle affects the surface roughness of the formed parts.

By controlling tool rotations, Eyckens et al. [5] found that the deformation of the metallic part during SPIF is linked to the friction effects caused by the tool tip radius. To obtain a better understanding of the friction mechanism between the tool and the forming of aluminum parts, Lu and co-workers [6] developed a stress analysis model that was used to confirm the significance of friction effects in the SPIF process due to forming forces, surface roughness, and part formability. Silva et al. [7] adapted the Taylor equation for the SPIF process and measured the wear on the tool. They concluded that higher spindle rotation speed improves the surface roughness of the formed component with increasing tool tip wear. Sornsuwit and Sittisakuljaroen [8] investigated how the surface roughness and part formability are affected by applying lubricants during the SPIF process and found that the tool tip, forming depth, and lubricant tribological properties influence the surface roughness of the specimen.

To understand how surface roughness and hardness are modified during the SPIF process, Azevedo et al. [9] investigated the influence that the type of lubricant has on the metallic aluminum part surface, while Diabb et al. [10] studied the performance that vegatable oils reinforced with SiO_2_ nanoparticles has during the SPIF of Al6061 metallic parts. Using Stribeck curves, they found an improvement on surface roughness and friction attained by the aluminum samples when reinforced lubricants were used. To investigate the impact that tool roughness has on controlling sheet metal forming, Sigvant et al. performed sheet metal forming simulations using a new a friction model with AutoForm^plus^, finding that the tool surface roughness plays an important role in sheet metal forming [11]. Slota and coworkers concluded that the surface properties of a Spifed metallic specimen is affected by the resulting residual stresses that cause large surface strains that lead to the orange peel phenomenon [12,13]. Recently Najm et al. studied how different SPIF parameters affect the hardness of an aluminum alloy specimen by considering coolant oil and different grease types. They found that if the specimen hardness needs to be increased, coolant oil must be used as a lubricant in addition to high feed rates and tool speeds [14].

However, none of the above articles addressed the effects that lubricant tribological properties and SPIF parameters such as vertical step size, wall angle, and tool tip radius could have on the surface roughness of the SPIFed parts. Therefore, this article focuses on investigating the influence that lubricants such as mineral oil, sunflower oil, and soybean oil have on the friction, wear, and along forces of AA1100 alloy metallic parts produced with the SPIF process by plotting the SPIFed Stribeck curves considering friction, wall angle, and forming depth. The advantages of determining the SPIFed Stribeck curves as a function of the manufacturing parameters and the impact of the lubricants are also studied. At the end of the paper, some effects that occur during SPIF are investigated by performing experimental characterizations on the formed surface samples using energy dispersive spectroscopy (EDS), a scanning electron microscope (SEM), confocal microscope measurements, and Fourier-transform infrared spectroscopy (FTIR).

The organization of this article is as follows: Section 2 gives an overview of SPIF process parameters and the lubricant properties used to form the pyramid shape of the metallic samples. Section 3 focuses on the tests performed to determine the friction and wear in the aluminun samples. The SPIFed Stribeck curves that arise by considering the fabrication parameter values and the lubricant tribological properties are also plotted. Experimental characterizations of the surface sample using EDS, SEM, confocal microscope measurements, and FTIR analysis obtained from the mineral and vegetable oils before and after the SPIF processing of the metallic parts are discussed in Section 4. Finally, Section 4 focuses on the conclusions of the article findings.

## 2. Materials and Experimental Tests

### 2.1. Materials

AA1100 alloy sheets (Aceros Levinson, Monterrey, México) trimmed with an abrasive water jet machining process to the size the of 150 mm × 150 mm with an initial averaged thickness value of 1.5 mm were used. Four different lubricants purchased from the AGYDSA Company (Zapopan, Mexico) were used: paraffinic mineral oil from FORKISA Company (Monterrey, México), sunflower, soybean and corn vegetable oils. The aluminum sheets were SPIFed using two hardened steel hemisphere tools with a tool-tip diameter of 5 mm and 10 mm. During the SPIF process of the metallic specimens, we added 10 mL of oil into the sample effective area of 120 cm × 120 cm. To reduce the friction effects between the tool and the SPIFed material specimens, a free tool rotation was considered. Thus, the wear, friction coefficient, and surface roughness effects were quantified as a function of the lubricant properties provided by the supplier such as viscosity, density, and the percentage concentration of fatty acid composition listed in Table 1.

### 2.2. SPIF Process

**Figure 1:**
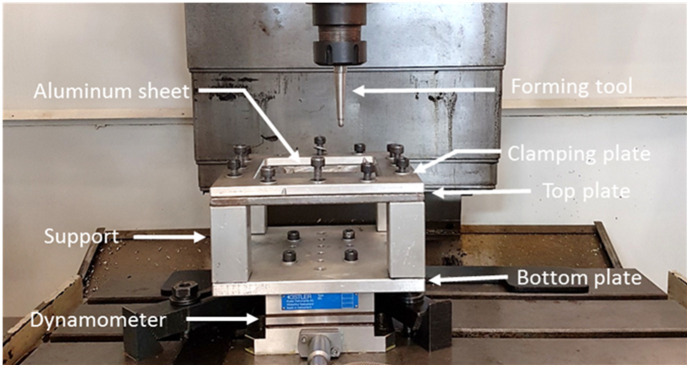
SPIF forming fixture mounted on a Kryle CNC milling center.

Figure 1 shows a fixture system composed of a support system, top plate, clamping plate, four rectangular supports, and a lower plate the was bolted onto the dynamometer measurement devices. The metallic sheet with the dimensions of 150 mm × 150 mm was placed between the clamping and top plates with workable area of 120 mm × 120 mm, as shown in Figure 1. The fixture system was mounted on a Kryle CNC 3-axis vertical machining center (Hemaq, Monterrey, México) equipped with a Kistler dynamometer type 9257B (Kistler Instruments, Monterrey, México), a charge amplifier, and an acquisition system.

**Figure 2:**
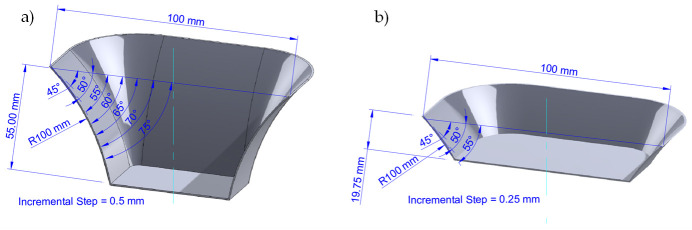
Pyramid shape vertical step sizes used to manufacture the aluminum sheets: (**a**) isometric view of frustum of a square base pyramid with an incremental vertical step size of 0.5 mm and 55 mm of sample height; (**b**) isometric view of frustum of a square base pyramid with an incremental vertical step size of 0.25 mm and 19.75 mm of sample height.

All of the metallic samples were formed following a pyramidal frustum with a square generatrix, a variable wall angle for each depth [15,16], and an incremental vertical step size of 0.25 mm and 0.5 mm, as shown in Figure 2. As expected, spring-back occurred to recover the metallic sheet, which produced some shape errors in the horizontal and vertical distance between the planned and final part geometry. Since the aim of the article is to investigate lubricant effects on the surface of the formed parts linked to manufacturing conditions, we do not elaborate any further on this but refer the intersted reader to Reference [17] in which a two-step incremental forming process was used to decrease shape errors.

### 2.3. Surface Roughness Profiles

**Figure 3:**
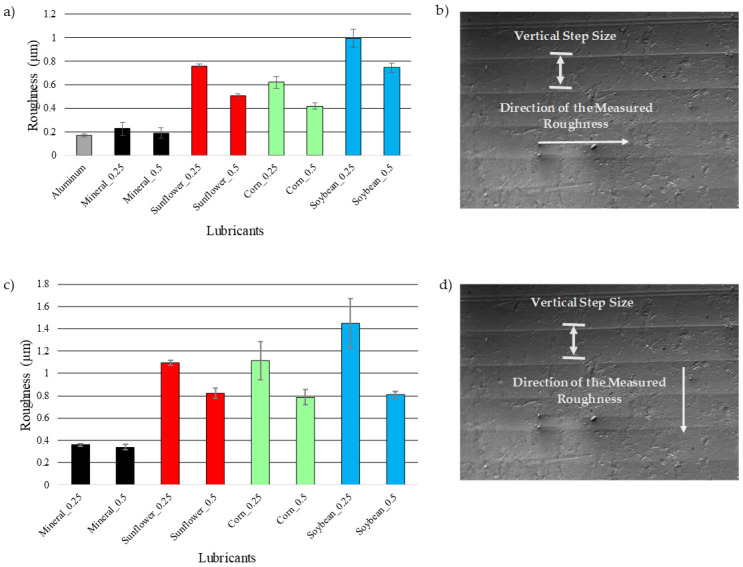
(**a**) Sample surface roughness (*Ra*) measured values for incremental steps of 0.25 and 0.5 mm; (**b**) sample surface roughness measured in the direction of the tool passes (tool-tip motion along the sample surface); (**c**) samples surface roughness (*Ra*) measured in the perpendicular direction to the passes for incremental steps of 0.25 and 0.5 mm; (**d**) surface roughness measured in the perpendicular direction to the tool passes.

In order to evaluate the effects that the step depth size and the tool tip diameter have on the surface sample roughness [18,19,20], after the samples were formed and unclamped from the machine fixture, ten roughness tests were performed on each SPIFed specimen using an Alicona Infinite Focus microscope (Bruker Alicona, Itasca, IL, USA) with a filter *L*_c_ = 80 µm. The average roughness values were obtained following the norm ISO 4288 and using the AutomationManager apparatus. The measured mean roughness (*Ra*) of the aluminum samples for the different vertical step size and semispherical tools used during the sample SPIF process are summarized in Figure 3. The aluminum bar indicates the measured surface roughness of the metallic samples. One can see from Figure 3a,c that mineral oils provide the best surface finish when compared to the values obtained from vegetable oils. Note that soybean oil produces the worst surface roughness finish when we consider a vertical step size of 0.25 mm and a tool tip with a 5 mm diameter.

### 2.4. Wear on Fabricated Samples

**Figure 4:**
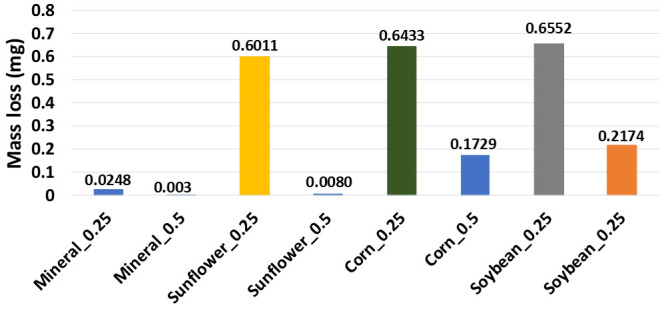
Mass loss versus different oils for incremental step of 0.5 and 0.25 mm recorded by using an Ohaus PA323 precision analytical balance.

Figure 4 shows experimental data related to the mass loss of the different material samples recorded after their forming during the SPIF process with the use of vegetable oil-bearing materials. Once more, mineral oil exhibits the least amount of wear when compared to those of the vegetable oil lubricants, except for sunflower oil. In fact, this vegetable oil lubricant produces the lowest sample wear values when a 10 mm tool tip and 0.5 mm step size are selected for the SPIF fabrication process of the aluminum sheets. Therefore, it can be concluded that the tool tip and sheet friction in the specimens impact formability. Furthermore, the tool size, the vertical step size, and the oil lubricant have a strong influence on the surface finish of the specimens produced by the SPIF process.

**Figure 5:**
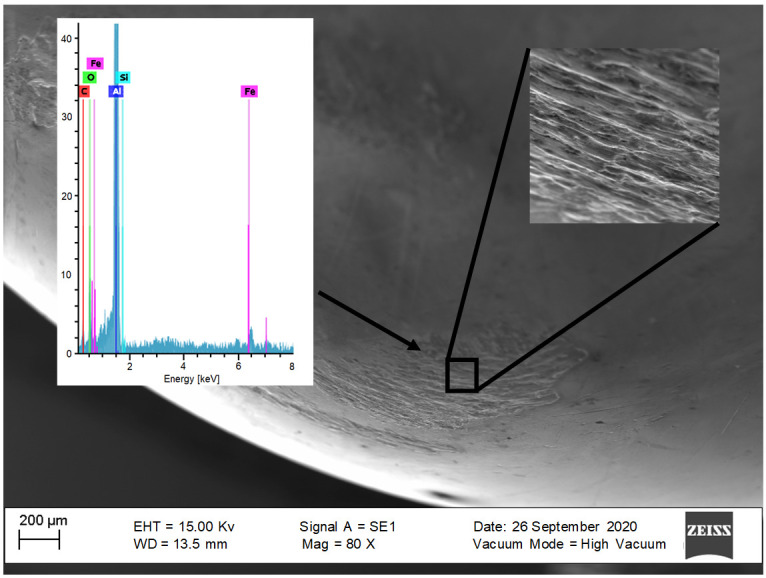
SEM–EDS images of the sample surface formed by a 10 mm tool tip and a 0.5 mm vertical step size with soybean oil lubricant.

To quantify the degree of debris particles during the SPIF process of the aluminum sheets, experimental measurements were performed with a scanning electron microscope (SEM) and energy dispersive spectroscopy (EDS). Figure 5 illustrates aluminum traces embedded on the 10 mm tool/punch tip when soybean oil was used during the SPIF process. This image confirms that the mechanism of adhesive wear is predominant in lubrication with soybean oil during the SPIF process. This phenomenon occurs because of the localized high pressure between the punch and the metallic sheet surface.

**Figure 6:**
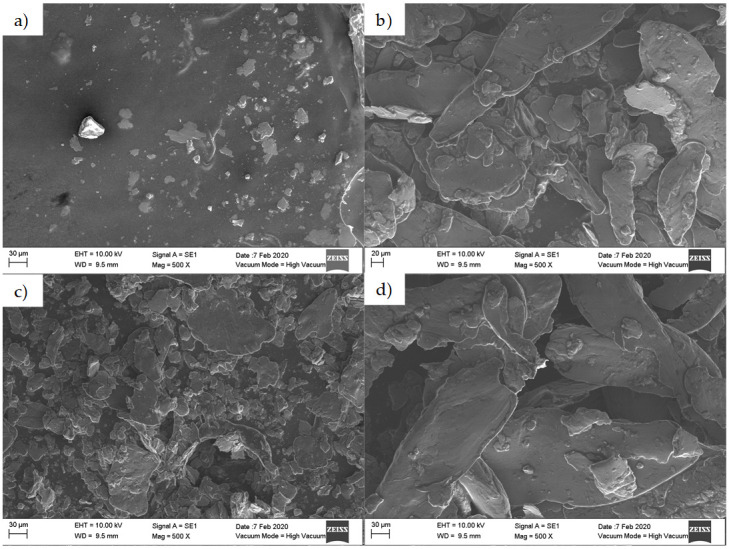
SEM-EDS images of debris particles detached from the surface of the aluminum sheets. Here the sample was formed by considering a step size of 0.5 mm and a 10 mm diameter tool tip: (**a**) mineral, (**b**) sunflower, (**c**) corn, and (**d**) soybean oils.

**Figure 7:**
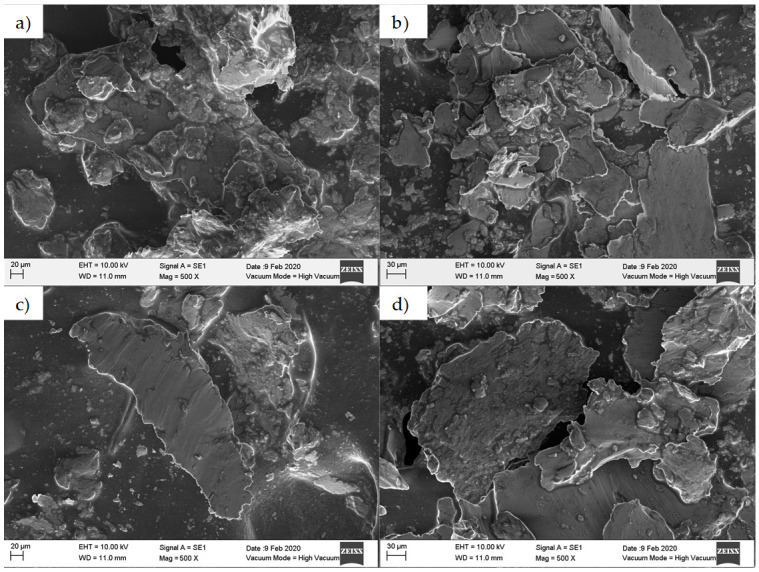
SEM–EDS images of debris particles detached from the surface of the aluminum sheets. Here the sample was formed by considering a step size of 0.25 mm and a 5 mm diameter tool tip: (**a**) mineral, (**b**) sunflower, (**c**) corn, and (**d**) soybean oils.

To further assess wear effects, the debris particles obtained from the vegetable oil tests were separated by means of vacuum filtering using Büchner flasks and polypropylene filters of 0.45 µm. Figure 6 and Figure 7 illustrate the morphology of the debris particles collected from the mineral and vegetable oils. Notice that aluminum flakes with different sizes were detached from the aluminum sheets when the soybean and corn oil lubricants were used.

**Figure 8:**
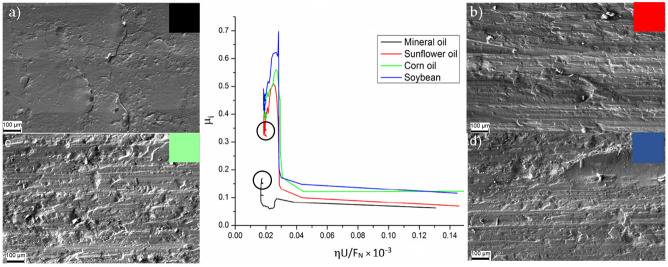
The SPIFed Stribeck curves and wear morphology of the sheet surface using SEM: (**a**) mineral, (**b**) sunflower, (**c**) corn, and (**d**) soybean oils for an incremental step of 0.5 mm and a 10 mm tool diameter.

**Figure 9:**
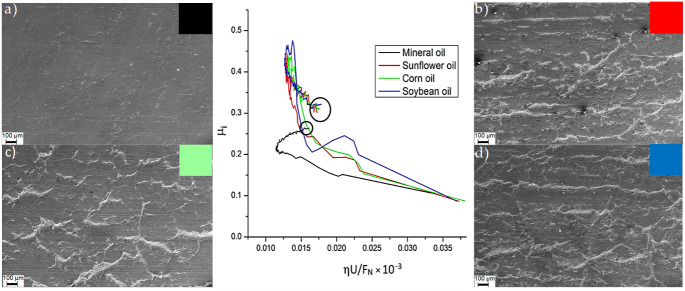
SPIFed Stribeck curves and wear morphology of the sheet surface using SEM: (**a**) mineral, (**b**) sunflower, (**c**) corn, and (**d**) soybean oils for an incremental step of 0.25 and a 5 mm tool diameter.

The SPIFed Stribeck curves and the worn surfaces of the aluminum samples produced with the step values of 0.25 mm and 0.5 mm are shown in Figure 8 and Figure 9, respectively. Examination of the worn surface of the metallic material samples indicates that vegetable oils increase surface roughness. If sliding friction occurs, then the material is detached from the aluminum sheet and is embedded into the tool tip surface resulting in the development of lines during SPIF process [5]. Notice from Figure 8 and Figure 9 that when the surfaces of the sample are lubricated with vegetable oils, they develop continuous wear grooves and material removal along the surface [21,22]. This condition is more evident for small vertical step size and tool tip diameter values. The SPIFed Stribeck curves and SEM images in Figure 8 and Figure 9 show material removal from the metal surface by a tearing mechanism along the tool moving path. This condition increases the metal surface roughness and the friction coefficient values (*µ*_i_). The experimental results obtained here agree with the observations of Le et al. [23] since their findings suggest that the use of smaller tool tip diameters could be more easily penetrated into the surface sheet, detaching material chips.

### 2.5. Fourier-Transform Infrared (FTIR) Measurements

**Figure 10:**
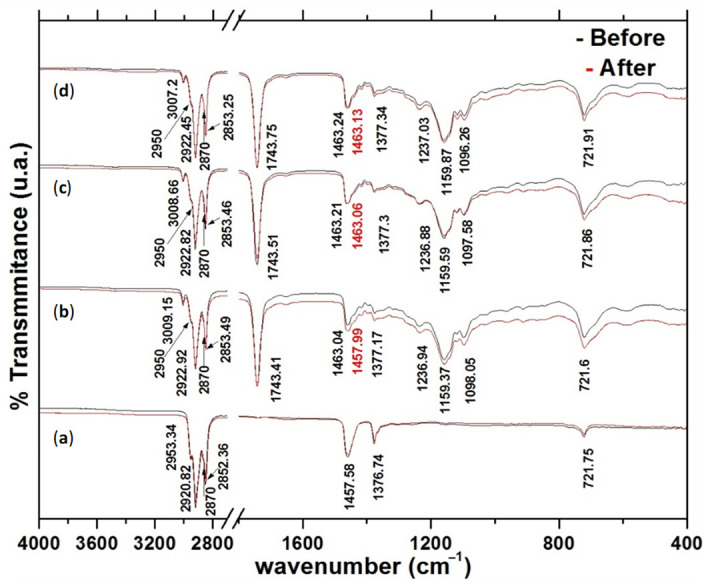
Oil spectrograms: (**a**) mineral oil, (**b**) soybean, (**c**) corn and (**d**) sunflower; before and after SPIF process.

Figure 10 shows Fourier-transform infrared (FTIR) spectrograms obtained from the mineral and vegetable oils before and after the SPIF processing of the metallic parts. The spectrogram of the mineral oil shown in Figure 10 exhibits the same characteristic vibration bands before and after the fabrication of the metallic parts. The agreement of these vibration bands indicates that a low chemical affinity of mineral oils with other substances exists [24].

In fact, these spectrograms in Figure 10 indicate that the main components (fatty saturated acids, fatty monounsaturated, and polyunsaturated acids) of the virgin vegetable oils are quite similar [25,26]. However, after the SPIF processing of the metallic sheets, the spectrograms of the vegetable oils show lightly batochromic shifts in the vibration band at 1463 cm^─1^, with the highest one occurring in soybean oil from 1463 to 1458 cm^−1^. Therefore, it can be concluded that when vegetable oil lubricants are subjected to pressure and friction forces as well as to metal impurities, these effects could favor isomeric changes allowing for a sigmatropic rearrangement in their chemical molecular structure [26]. It is believed that the small difference in the vibrational bands of the vegetable oil lubricant spectrograms occurs because of a physical adsorption process with light batochromic shift.

## 3. Results and Discussions

### 3.1. Determination of Friction Coefficients and Wear of the Aluminum Samples

Wear tests were performed considering the lubricated sliding conditions on the surface of the SPIFed metallic aluminum sheets. All tests were conducted over 9 min, and the mass lost by the specimens was recorded with the gravimetric method using an Ohaus Latinoamerica PA323 precision analytical balance [Mexico City, México]. The tested sliding distances of each material sample was 2000 m.

**Figure 11:**
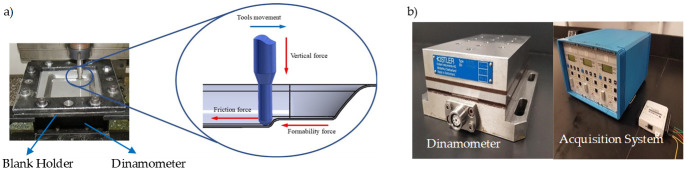
(**a**) Forces acting during SPIF process. (**b**) Kistler dinamometer model 9257B, Kistler amplifier system model 5010, and National Instrument NI USB 6008 data acquisition system.

For illustrative purposes, the collected SPIF forming forces experienced by the specimens are illustrated in Figure 11. Recognizing the difficulties of measuring the friction coefficients because of the recorded horizontal force containing the friction and forming forced components, an alternative strategy has to be followed in which a friction indicator *µ*_i_ is used to quantify the friction condition effects. This friction indicator is determined using the following equation [6,27]:

(1) $$\mu_{i}=\frac{F_{f}+F_{form}}{\left|F_{N}\right|}=\frac{F_{a}}{\left|F_{N}\right|},$$

where *F_f_* is the friction force, *F*_form_ is the forming force, *F_a_* represents the magnitude of the along force, and *F_N_* provides the normal contact force magnitude.

### 3.2. Determination of the Forming Forces and Friction Coefficients

**Figure 12:**
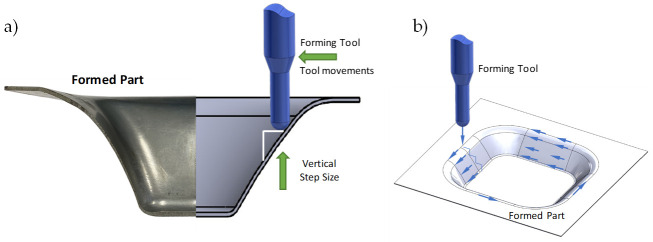
(**a**) Toolpath of vertical step size, and (**b**) schematic of the forming cone shape, tool path, and the along force *Fa*.

To determine the forming forces and the friction coefficients that the aluminum samples experienced during the SPIF process, a series of tests with different oils were carried out. Figure 12a,b show the toolpath on the walls of the forming sample.

**Figure 13:**
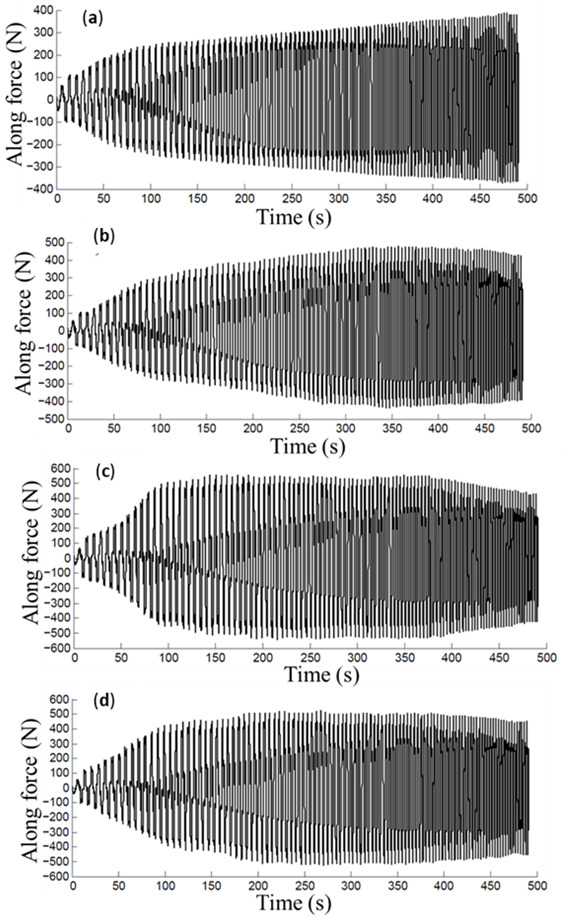
Along force magnitude curves recorded during the aluminum sheet manufacturing process considering the following lubricants: (**a**) mineral, (**b**) sunflower, (**c**) corn, and (**d**) soybean oils. Here *∆z* = 0.25 mm with a 5 mm tool tip diameter.

**Figure 14:**
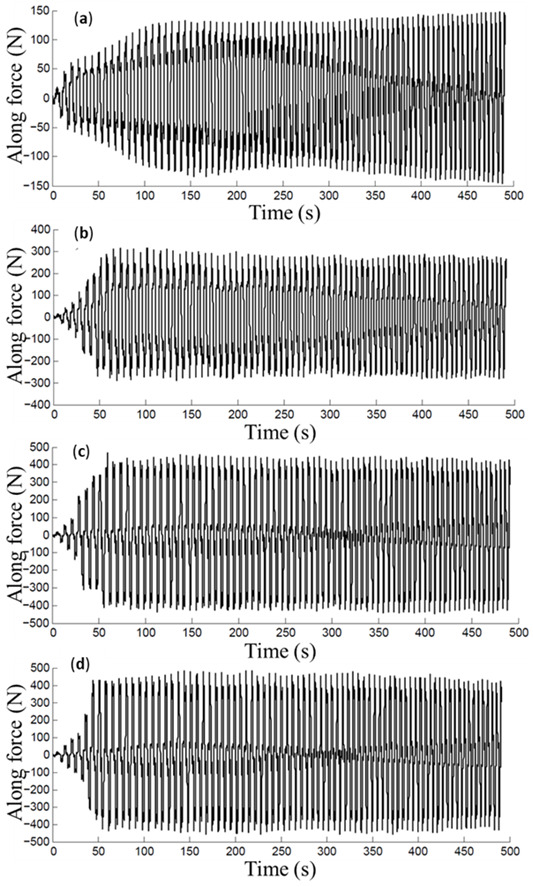
Along force magnitude curves recorded during the aluminum sheet manufacturing process considering the following lubricants: (**a**) mineral, (**b**) sunflower, (**c**) corn, and (**d**) soybean oils. Here *∆z* = 0.5 mm with a 10 mm tool tip diameter.

The along forming force magnitude curves of the metallic sheets are shown in Figure 13 and Figure 14. Notice from Figure 5 and Figure 6 that the maximum along force magnitudes are generated when the samples are SPIFed with ∆*z* = 0.25 mm with a 5 mm tool tip diameter. From Figure 13 and Figure 14, it can be concluded that the tool tip diameter and the vertical step size affects the along forming force values. These findings coincide with previously reported results by Duflou [15].

**Figure 15:**
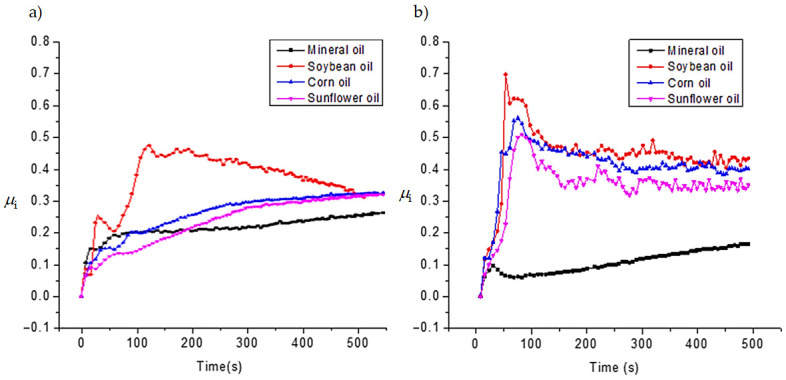
Friction coefficients attained using incremental step size of: (**a**) 0.25 and (**b**) 0.5 mm.

The curves of Figure 15 show the influence that the mineral and vegetables oils had in the variation of the friction coefficient values during the sample forming process. Here, the soybean and corn oils attained the highest forces and friction coefficients when compared to those of sunflower oil. These experimental findings agree with the conclusions of Bart et al. [28], where it was found that vegetable oils presented poor oxidative stability, which favors the change of the physical properties of the oil such as viscosity, acidity, among others. Therefore, the modification of these physical properties and the detachment of aluminum oxide from the AA1100 aluminum alloy forming samples could lead to increasing forces and friction coefficient values.

### 3.3. The SPIFed Stribeck Curve

**Figure 16:**
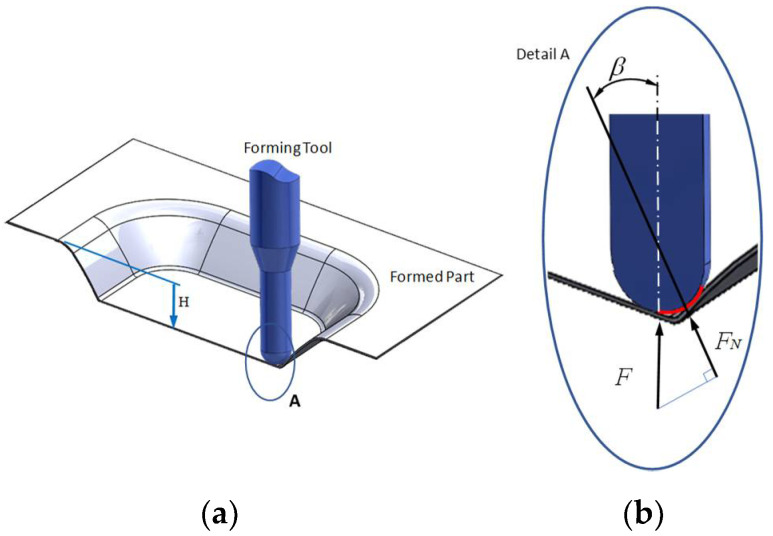
SPIF tool tip force components: (**a**) isometric view of the formed part and the forming tool; (**b**) isometric sectional view that shows the tool tip forming force components during Spif process.

In this section, the SPIFed Stribeck curves are plotted to capture the behavior of the lubricants under hydrodynamic, mixed, and boundary regimes during manufacturing process of the aluminum sheets using the equation *S* = *ηU*/*F_N_*, where *U* is the mean speed, *η* is the dynamic viscosity assumed to have a constant value, and *F_N_* describes the forming normal force [10,29]. Here, *U* depends on the distance travelled of the tool path along the surface sample and on the time span needed to complete the tool cycle for each vertical forming depth value. Thus, *U* can be found from the following equation: *U* = *ωd*/(2π), where *d* is the tool travel distance at each forming depth value, *ω* is the frequency value related to the time span needed to complete one tool cycle. Normal force is found by considering the projection of the vertical force acting on the tool along the normal angle *β* to the line of sliding contact in the plane of the aluminum surface sample, as illustrated in Figure 16.

**Figure 17:**
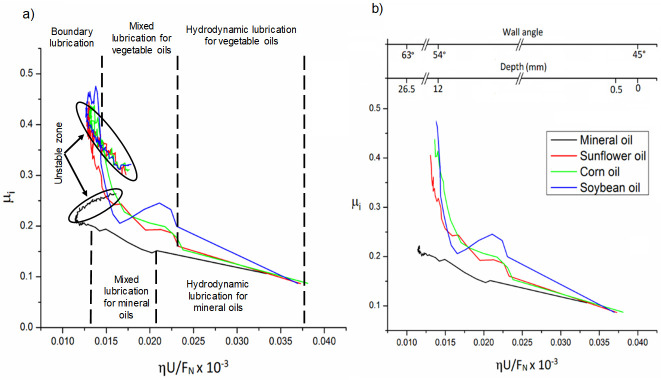
The SPIFed Stribeck curves considering a vertical step size of 0.5 mm and a 10 mm tool tip diameter: (**a**) Stribeck curves during the SPIF of aluminum sheets using mineral oil, sunflower, corn and soybean oils. (**b**) Stribeck curves as a function of SPIF wall angle and step depth. Experimental tests were performed at room temperature.

Figure 17 shows the computed SPIFed Stribeck curves for each lubricant plotted by considering a vertical step size of 0.5 mm and a tool tip diameter of 10 mm. Note that the SPIFed Stribeck curve of the mineral oil has the lowest friction coefficient values. In addition, the SPIFed Stribeck mineral oil curve does not show abrupt changes during lubrication regimens, which is the case for the soybean oil curve, which shows a linear friction indicator variation for high values of *ηU*/*F_N_*. The lubricant fluid film then becomes thinner, and the metal–metal contact starts to occur (mixed and boundary lubrication regimes). The corn and sunflower SPIFed Stribeck oil curves exhibit similar behavior. In both cases, the boundary lubrication regime is found to start at about *S* = 0.015 × 10^−3^ m^−1^. It is also interesting to observe from Figure 17, that for this forming stage, the soybean SPIFed Stribeck curve almost reaches the maximum friction coefficient value of 0.5 (Blue solid line). It can also be noticed from Figure 17, that for *S* close to the value of 0.015 × 10^−3^ m^−1^, the mineral oil (black solid line) causes a friction coefficient value that does not exceed of 0.27. Furthermore, for certain forming step depth and wall angle values, the contact interface between the surface sample and the tool’s hemi-spherical tip moves through the boundary film lubrication regime (asperity contact), reaching the maximum friction coefficient magnitude values and then, the friction coefficient values start to decrease because of the SPIF bending moment effects [30].

**Figure 18:**
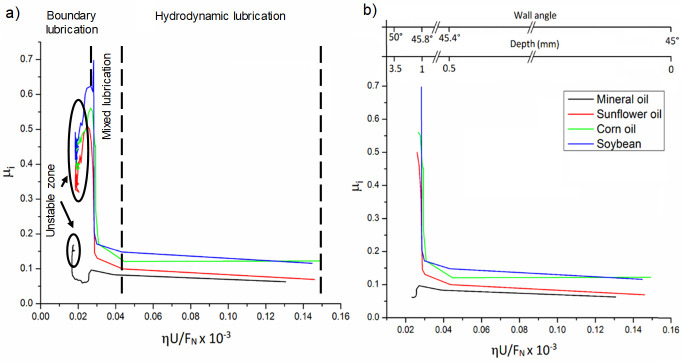
SPIFed Stribeck curves plotted considering a vertical step size of 0.25 mm and a 5 mm tool tip diameter. (**a**) Stribeck curves during the SPIF of aluminum sheets and (**b**) Stribeck curves plotted considering the SPIF wall angle and forming step depth values. All data were collected by performing experimental tests at room temperature.

Figure 18 shows SPIFed Stribeck curves plotted considering the process wall angle and the forming depth values. These curves were obtained by setting the vertical step to 0.25 mm and using a 5 mm tool tip diameter. From Figure 17 and Figure 18, one can see that for high values of *ηU*/*F*_N_, the friction coefficient linearly descends because of the film lubrication. When the normal force *F*_N_ increases or the lubricant viscosity or velocity *U* decreases, the magnitude of *ηU*/*F*_N_ becomes smaller. This is an indication of oil film thickness reduction with an increase of the friction coefficient due to the degree of debris particles during the SPIF processing of the aluminum sheets. For smaller values of *ηU*/*F*_N_, the tool tip with aluminum sheet (metal-to-metal) contact starts to occur and thus, the friction coefficient tends to increase sharply (boundary lubricant regime) [31]. In both Figure 17 and Figure 18, the SPIFed Stribeck curves of the lubricant oils exhibit hydrodynamic and mixed lubrication regimens in the region of 0.035 × 10^−3^ m^−1^ to 0.14 × 10^−3^ m^−1^. Here, the boundary regime takes place for values of *ηU*/*F*_N_ less than 0.03 × 10^−3^ m^−1^.

## 4. Conclusions

The effect that petroleum and vegetables oil-based lubricants have on the friction coefficient, wear, along forces, and surface roughness of metallic sheets manufactured using the SPIF process has been investigated by considering the vertical step size, wall angle, and semispherical tool tip diameter values. It was found that during the SPIF process metallic sheets, soybean and corn oils attained the highest friction, along forces, roughness, and wear. These results are confirmed by SPIFed Stribeck curves of the soybean and corn oils in which these lubricants exhibited small hydrodynamic regime span for an increasing sample forming process. From these SPIFed Stribeck curves, it is possible to identify the values of *ηU*/*F*_N_ from which the lubrication regime changed from a mixed film to the boundary regimes when the wall angle and SPIF depth values changed. Additionally, the experimental characterization of the samples surface with SEM show that the worn surface of the metallic material samples produced with vegetable oils have increased surface roughness when compared to those produced with mineral oils. Based on the surface roughness measurements, it has been found that soybean oil produces the worst surface roughness finish in the perpendicular direction to the tool passes, with a value close to *Ra* = 1.45 μm for a vertical step size of 0.25 mm and a 5 mm tool tip diameter. These findings are confirmed by plotting SPIFed Stribeck curves for the soybean and corn oils that show small hydrodynamic span regime for increasing sample step-size forming process.

The FTIR experimental characterization of vegetables oils used during the SPIF process of the metallic parts show that when vegetable oil lubricants are subjected to pressure and frictional forces and metallic impurities, isomeric changes occur that allow sigmatropic rearrangement in their chemical molecular structure. However, less wear occurs in parts produced with mineral oil due to the absence of functional groups, low chemical affinity, and more efficient packaging of the linear hydrocarbonated chain. Therefore, one can conclude that the friction between the tool and the metallic sheet surface plays an important role in material deformation. Furthermore, the tool tip diameter, wall angle, depth, vertical step size, and tribological properties of the oil lubricant have a strong influence on the surface-finishing of parts produced by the SPIF process.

## Figures and Tables

**Table 1:** Some lubricant properties and the percentage concentration of fatty acid components in the oils. Adapted from Ref. [10] with Copyright Elsevier License Number 5100891486653.

Oil	Density (g/cm^3^)	Viscosity (Pa-s)	Saturated %	Monounsaturated %	Polyunsaturated %
Mineral	0.90	0.0408	–	–	–
Sunflower	0.92	0.0486	11	20	69
Corn	0.91	0.0466	13	25	62
Soybean	0.90	0.0452	15.2	22.8	60.9

## Data Availability

Data available are upon request due to restrictions such as privacy. The data presented in this study are available upon request from the corresponding author.
